# Abortion Stigma and Its Relationship with Grief, Post-traumatic Stress, and Mental Health-Related Quality of Life After Abortion for Fetal Anomalies

**DOI:** 10.1089/whr.2021.0027

**Published:** 2022-03-28

**Authors:** Jennifer Kerns, Morgan Cheeks, Arianna Cassidy, Geffan Pearlson, Biftu Mengesha

**Affiliations:** ^1^ Division of Zuckerberg San Francisco General, Department of Obstetrics, Gynecology, and Reproductive Sciences, University of California San Francisco, San Francisco, California, USA.; ^2^ University of California San Francisco School of Medicine, San Francisco, California, USA.; ^3^ Division of Maternal-Fetal Medicine, Department of Obstetrics, Gynecology, and Reproductive Sciences, University of California San Francisco, San Francisco, California, USA.

**Keywords:** abortion stigma, fetal anomalies, post-abortion psychological health, pregnancy termination

## Abstract

**Background:**

Despite the prevalence of abortion stigma in the United States, few studies have examined the relationship between stigma and psychological well-being postabortion among women who undergo abortion for fetal anomalies.

**Materials and Methods:**

We conducted a cross-sectional study of women who underwent second-trimester abortion for pregnancy complications to assess the association between abortion stigma and psychological outcomes. We asked women to retrospectively report self-judgment and perceived community condemnation at the time of their abortion and evaluated present-day grief, post-traumatic stress, and self-reported mental health. We recruited participants using Facebook, Craigslist, and other public online forums. We used multivariable linear regression to evaluate relationships between abortion stigma and psychological outcomes. In adjusted models, we controlled for covariates that were associated with the outcome at a level of *p* < 0.1.

**Results:**

Adjusted models, including 80 women, revealed that higher self-judgment at the time of abortion was significantly associated with increased postabortion grief (*β* = 2.5 and *p* = 0.02). Self-judgment was not associated with statistically significant differences in post-traumatic stress or mental health. There was no association between perceived community condemnation and psychological outcomes.

**Discussion:**

Abortion stigma may be associated with increased postabortion grief, but does not appear to be associated with differences in post-traumatic stress or mental health. Investigating how different preprocedure counseling methods can impact self-judgment might inform future interventions aimed at improving psychological outcomes postabortion.

**Implications for Practice and/or Policy:**

Abortion providers should consider that women who display signs of self-judgment may be at higher risk for increased grief after pregnancy termination for fetal anomalies or maternal complications.

## Introduction

Abortion stigma is a widely recognized phenomenon, described as a negative attribute ascribed to women who seek to terminate a pregnancy, which marks them, internally or externally, as inferior to ideals of womanhood.^1^ Multiple large studies conducted in the United States (US) have established that a majority of people who have had an abortion perceive stigma related to the abortion.^2,3^ Two-thirds of women who have had an abortion feel they would be looked down upon as a result and more than half felt they needed to hide their abortion from friends and family.^3^

Abortion stigma can also affect women undergoing abortion for fetal anomalies or maternal complications. Major congenital fetal anomalies are diagnosed in ∼3%–4% of pregnancies, typically in the second trimester, and poor fetal prognosis often results in a woman's decision to undergo abortion.^1,3–6^ Maternal indications necessitating abortion make up an estimated 4% of US abortions.^7^ Abortion for fetal anomalies and maternal complications constitutes a unique subset of patients where an unfavorable diagnosis prompts a decision to end the pregnancy.

Those terminating for unexpected fetal or maternal diagnoses report similar experiences with decision-making around the termination method and psychological recovery post-termination. Moreover, because these terminations are more likely to occur in the second trimester when pregnancies are more public, it is possible that abortion stigma may play a larger role.^8.9^

In studies of women undergoing abortion for any reason (*i.e.*, not specifically for fetal anomaly), abortion stigma is associated with poor psychological outcomes, including grief, post-traumatic stress, anxiety, and depression postabortion.^10,11^ In fact, a recent study found that abortion stigma was associated with four times higher odds of psychological distress postabortion.^2^ Psychological distress, specifically post-traumatic stress and depression, has also been documented after termination for fetal anomalies.^12,13^

Higher gestational age^12^ and decisional conflict were associated with more grief after termination, whereas acceptance and positive reframing were protective against grief.^14,15^ Notably, the authors describe termination for fetal anomaly as a traumatic event experienced by individuals within the sociopolitical–legal environment^14^; in the US, this environment is marked by divisions between those supporting and opposing abortion and lays the groundwork for widespread abortion stigma. The specific role that stigma plays in the relationship between abortion for fetal anomalies and psychological distress is unknown.

Most current literature on abortion stigma either outlines a conceptual framework or examines associations between demographic characteristics and abortion stigma.^1,3,16^ One recent framework for abortion stigma examines self-judgment and perception of community condemnation as key components of abortion stigma.^16^ The experience of stigma is particularly important to explore in women seeking abortion for fetal anomaly given that this diagnosis is often devastating for women and may put them at increased risk for negative psychological outcomes.^12,17,18^

Furthermore, much of the literature on stigma does not distinguish between reasons for abortion, and restrictive abortion laws that allow abortion for fetal anomalies or maternal complications suggest that an abortion for these diagnoses may be more socially acceptable. Understanding whether or not these aspects of abortion stigma contribute to postabortion psychological outcomes specifically in women having abortions for pregnancy complications could improve counseling efforts to minimize psychological distress.

In this study, we examine how retrospective measures of self-judgment and perception of community condemnation at the time of abortion impact postabortion grief, post-traumatic stress, and overall mental health in women who had an abortion for maternal or fetal complications in the second trimester. We felt that these two subscales were the most relevant for women undergoing abortion based on clinical experience and available literature.^19^

In previously published work, we find that women who terminate for fetal anomalies and/or maternal complications are generally unified with their partners and loved ones about their decision. We felt that worries about judgment and isolation would be less relevant in this context and thus only included self-judgment and perceived community condemnation in our data collection.^8,9^

## Materials and Methods

This study was approved by University of California, San Francisco Committee on Human Research.

We conducted a cross-sectional study in 2016 of English-reading women aged 18 years and older in the US who had ever undergone a second-trimester abortion for fetal anomalies or maternal complications. Only women who had their abortion between 2010 and 2015 were included in this analysis to reduce recall bias. We used REDCap, a secure online survey platform, to create and distribute our survey instrument and recruited participants using Facebook, Craigslist, and other public online forums. Because this was an exploratory study, we recruited a convenience sample and did not have a prespecified sample size.^20^

We collected data on pregnancy diagnosis, reproductive history, and type of abortion procedure (dilation and evacuation [D&E] vs. induction of labor). We assessed present-day demographics (such as income and education), decision satisfaction, depression, trait anxiety, post-traumatic stress, and grief. Finally, we asked women to reflect on the following items at the time of their abortion: mental health-related quality of life (HRQOL) in the 30 days before and following their abortion, shared decision-making (SDM-9), and abortion stigma, including self-judgment and perceived community condemnation.

We examined the relationship between abortion stigma at the time of the abortion and three psychological outcomes postprocedure: present-day grief, post-traumatic stress, and mental HRQOL in the 30 days following their abortion. We chose these as our primary outcomes because we wanted to narrow our analysis to experiences specifically related to the abortion. We measured grief using a modified version of the Perinatal Grief Scale, a 33-item scale assessing active grief, difficulty coping, and despair. Higher values correlate with higher levels of grief.^21^

We assessed postabortion post-traumatic stress using a modified version of the Impact of Event Scale, which evaluates intrusive experiences and avoidance of thoughts and images associated with the event. Items were modified to include language about pregnancy loss. For example, the item “My feelings about it are kind of numb” was modified to read “My feelings about the pregnancy loss are kind of numb.”^20^ Higher scores correlate with greater post-traumatic distress.^22^ To assess overall mental health, we used the mental health subscore of the Healthy Days Core Module (HRQOL-4) from the Centers for Disease Prevention and Control.^23^ This metric is the self-reported number of days that were affected by poor mental health in the 30 days postabortion.

The primary covariate of interest was abortion stigma, as measured by two subscales of the Individual-Level Abortion Stigma (ILAS) scale: self-judgment and perceived community condemnation.^16^ We calculated scores by summing item values and dividing by the total number of items. Scores for both subscales ranged from 0 to 4, with higher ILAS scores reflecting higher levels of stigma.^16^

The self-judgment items from the ILAS scale include asking participants how strongly they agree with the statements “I felt like a bad person,” “I felt confident I made the right decision,” “I felt ashamed about my abortion,” “I felt selfish,” and “I felt guilty” using a 5-point Likert scale of strongly disagree to strongly agree.

The perceived community condemnation items of the ILAS scale asked participants to report how many people in their community held the following beliefs: “Abortion is always wrong” and “Abortion is the same as murder,” with possible responses being no one, a few people, about half the people, many people, and most people.

Factor analysis for ILAS conducted by the scale developers showed high rates of internal consistency and reliability: self-judgment (*a* = 0.84) and perceived community condemnation (*a* = 0.78).^16^

To develop a list of possible covariates, we considered survey items that were unlikely to be in the causal pathway, but could be associated with our outcomes of interest. We considered the following variables as possible covariates: maternal age at the time of abortion, race and ethnicity, income, education level, geographic location (by US region), gestational duration at the time of abortion, type of abortion procedure (D&E or induction termination), SDM-9 for abortion method, satisfaction with the abortion method decision, depression, anxiety, and preabortion mental HRQOL.

Present-day SDM-9, satisfaction with the abortion method decision, depression, and trait anxiety were included as possible covariates because of their complex relationship with our primary outcomes and to allow our model to focus on the relationship between stigma and our primary outcomes. When developing this list, we drew on prior research demonstrating that preabortion mental health is predictive of postabortion depressive, anxiety, and stress symptoms.^24^

The SDM-9 scale measures the patient's desired involvement in decision-making, including how well the provider valued patient preferences.^25^ We calculated scores by summing responses and scaling scores to range from 0 to 100, with higher values indicating higher levels of SDM-9. We used a modified version of the Satisfaction with Decision (SWD) Scale, which includes six items that examine how a participant felt about the options presented by her provider and whether she thought her decision was consistent with her values.

We summed responses to produce overall SWD scores, which ranged from 6 to 30. Higher scores indicate greater SWD.^26^ We measured depression using the Patient Health Questionnaire 9 (PHQ-9), with higher scores indicating more severe depression.^27^ We measured trait anxiety using the State-Trait Anxiety Inventory Form Y-2, a 20-item scale assessing trait anxiety, with higher scores indicating higher levels of anxiety.^28^

Trait anxiety is “the stable tendency to attend to, experience, and report negative emotions such as fears, worries, and anxiety across many situations.” We included trait anxiety to help control for variation in how individuals report experiences after they occur.^29^ Finally, we measured preabortion mental health as the number of days in the 30 days preabortion that were affected by poor mental health.

We calculated descriptive statistics using means and standard deviations for continuous variables and proportions for categorical variables. All scale scores were normally distributed in our population and no outliers were identified. We developed a list of possible confounders *a priori* and conducted unadjusted and adjusted linear regression models between possible confounders and each of the psychological outcomes. We used separate models for self-judgment and perceived community condemnation because community condemnation may increase self-judgment.

Covariates were included in the final model if they were statistically significant in the bivariate analysis. We included covariates associated with the outcomes at a level of *p* < 0.1 in our adjusted models. Because SWD and SDM-9 are highly correlated,^20^ we only included the variable with greater effect size and statistical significance to avoid overfitting and remain conservative in our estimates.

We tested our final models for multicollinearity using correlation tables and variance inflation factors when necessary. No multicollinearity was identified, thus we did not make any further adjustments to our models. We conducted all analyses using Stata (version 15.1, College Station, TX). We obtained approval for this study from the Committee on Human Research at our home institution. Participants did not receive compensation as part of this study.

## Results

We received 245 survey responses. We excluded 39 because they did not consent, 44 people because they reported they had never had an abortion, and 42 people because their abortion took place more than 5 years before survey completion. We removed 33 people from our analysis because they did not complete any survey questions for any of the following items of interest: self-judgment, perceived community condemnation, grief, post-traumatic stress, and mental HRQOL.

An additional five participants were removed because they had completed less than half of the data of interest. Two people were removed because they underwent termination of pregnancy for maternal complications. A demographic comparison of these participants and those included in the analysis is presented in Supplementary Table S1.

We analyzed data for 80 respondents who met inclusion criteria and had complete data for the covariates and outcomes of interest. We used listwise deletion for any participants missing one or more components of covariates or outcomes requiring responses from multiple survey questions. Participants were mostly White, had an income >$90,000 per year, and had high levels of education (Table 1).

**Table 1.** Characteristics of Study Participants

Characteristic	*n* = 80
Age at termination, years, mean (SD)	35.5 (5.2)
Missing, *n* (%)	0 (0.0)
Race/ethnicity, *n* (%)	
White	70 (87.5)
Non-White	10 (12.5)
Missing	0 (0.0)
Annual income, *n* (%)	
>$90,000	54 (67.5)
<$90,000	26 (32.5)
Missing	0 (0.0)
Highest level of education, *n* (%)	
Less than high school	1 (1.3)
High school or GED	1 (1.3)
Bachelor's degree	39 (48.8)
Graduate degree	39 (38.8)
Missing	0 (0.0)
Urban–rural classification, *n* (%)	
Urban	27 (33.8)
Suburban	46 (57.5)
Rural	5 (6.3)
Missing	2 (2.5)
Insurance type at the time of the study, *n* (%)	
Private	79 (98.8)
Public	0 (0.0)
None	1 (1.2)
Missing	0 (0.0)
Gestational duration (in weeks) at abortion, mean (SD)	20.5 (3.8)
Missing, *n* (%)	0 (0.0)
Abortion procedure type, *n* (%)	
D&E	52 (65.0)
Induction of labor	20 (24.4)
Not specified	8 (10.0)
Region of US where abortion occurred, *n* (%)	
West	43 (53.8)
Northeast	13 (16.3)
Midwest	7 (8.8)
South	17 (21.3)
Missing	0 (0.0)
Nulliparous, *n* (%)	
Yes	31 (38.8)
No	48 (60.0)
Missing	1 (1.2)
Wait time from decision about procedure type to abortion, days, mean (SD)	4.9 (3.7)
Missing, *n* (%)	8 (10.0)
SDM-9^a^ score, mean (SD)	62.5 (27.1)
Missing, *n* (%)	8 (10.0)
SWD^b^ score, mean (SD)	22.6 (6.0)
Missing, *n* (%)	8 (10.0)
Self-judgment^c^ score, mean (SD)	1.7 (1.1)
Missing, *n* (%)	8 (10.0)
Community condemnation^d^ score, mean (SD)	1.4 (0.8)
Missing, *n* (%)	10 (12.5)
Anxiety (STAI)^e^ score, mean (SD)	43.9 (13.6)
Missing, *n* (%)	2 (2.5)
Depression (PHQ-9)^f^ score, mean (SD)	8.0 (5.6)
Missing, *n* (%)	6 (7.5)
Grief (PGS)^g^ score, mean (SD)	57.5 (13.2)
Missing, *n* (%)	1 (1.3)
Post-traumatic stress (IES)^h^ score, mean (SD)	24.3 (10.9)
Missing, *n* (%)	2 (2.5)
Mental health^i^ preabortion, days, mean (SD)	10.7 (10.3)
Missing, *n* (%)	6 (7.5)
Mental health^i^ postabortion, days, mean (SD)	24.6 (9.1)
Missing, *n* (%)	6 (7.5)

^a^ SDM-9, range = 0–100, higher scores indicate higher shared decision-making.

^b^ SWD, range = 6–30, higher scores indicate higher satisfaction.

^c^ Self-judgment, range = 0–4, higher scores indicate more self-judgment.

^d^ Community condemnation, range = 0–4, higher scores indicate more feelings of community condemnation.

^e^ STAI, range = 20–80, higher scores indicate more anxiety.

^f^ PHQ-9, range = 0–27, higher scores indicate more depression.

^g^ PGS, range = 19–95, higher scores indicate more grief.

^h^ IES, range = 0–55, higher scores indicate more post-traumatic stress or poorer coping.

^i^ Mental health (HRQOL), range = 1–30, higher scores indicate more days of activity affected by poor mental health.

D&E, dilation and evacuation; GED, General equivalency diploma; HRQOL, health-related quality of life; IES, Impact of Event Scale; PGS, Perinatal Grief Scale; PHQ-9, Patient Health Questionnaire 9; SD, standard deviation; SDM-9, shared decision-making; STAI, State-Trait Anxiety Inventory; SWD, Satisfaction with Decision; US, United States.

Approximately half of the respondents lived in a Western state and most lived in either urban or suburban areas. All participants had an abortion within 5 years of survey completion, and the vast majority terminated due to fetal complications rather than maternal complications.

In unadjusted analyses, higher self-judgment was associated with more grief, more post-traumatic stress, and more days affected by poor mental health (*β* = 7.4, 4.3, and 2.3, respectively; *p* < 0.05 for all). Higher perception of community condemnation was associated with more grief and post-traumatic stress (*β* = 6.1 and 5.2, respectively; *p* < 0.05 for all). Higher trait anxiety, income, and SWD scores and higher retrospective measures of SDM-9 at the time of abortion were independently associated with less grief postabortion (*p* < 0.05 for all).

Trait anxiety was positively correlated with grief, post-traumatic stress, and more days affected by poor mental health (*p* < 0.05). Higher levels of SDM-9 were associated with less present-day post-traumatic stress (*p* < 0.05). Poor preabortion mental health was associated with significantly more days of poor mental health in the 30 days following the abortion (*p* < 0.05) (Table 2).

**Table 2.** Unadjusted Linear Regression of the Association Between Patient Characteristics and Postabortion Grief, Coping, and Mental Health-Related Quality of Life

Independent variable	Dependent variable	Regression coefficient (95% CI)	*p*
Older age at termination	Grief^a^	−0.5 (−1.1 to 0.1)	0.08
	Post-traumatic stress^b^	−0.3 (−0.7 to 0.2)	0.30
	Mental health^c^	0.1 (−0.3 to 0.5)	0.56
White/Caucasian	Grief	1.1 (−7.9 to 10.0)	0.81
	Post-traumatic stress	−1.6 (−9.0 to 5.7)	0.67
	Mental health	3.1 (−3.1 to 9.2)	0.33
Annual income >$90,000	Grief	−6.5 (−12.7 to −0.4)	**0.04**
	Post-traumatic stress	−2.9 (−8.1 to 2.3)	0.27
	Mental health	−2.5 (−7.0 to 2.0)	0.28
Graduate degree	Grief	−4.7 (−10.6 to 1.2)	0.12
	Post-traumatic stress	−5.0 (−9.8 to −0.2)	**0.04**
	Mental health	5.4 (1.4 to 9.5)	**0.01**
Living in an urban area	Grief	2.6 (−2.6 to 7.8)	0.33
	Post-traumatic stress	2.2 (−2.2 to 6.5)	0.32
	Mental health	−2.0 (5.9 to 2.0)	0.32
Termination in a state with restrictive termination laws	Grief	2.4 (−4.3 to 9.2)	0.47
	Post-traumatic stress	1.9 (−3.7 to 7.6)	0.50
	Mental health	0.8 (−4.0 to 5.5)	0.75
Higher gestational age at termination	Grief	−0.2 (−0.9 to 0.6)	0.69
	Post-traumatic stress	−0.2 (−0.8 to 0.5)	0.60
	Mental health	0.3 (−0.2 to 0.9)	0.22
Having a D&E procedure	Grief	2.4 (−4.6 to 9.4)	0.49
	Post-traumatic stress	4.8 (−1.0 to 10.6)	0.10
	Mental health	−3.7 (−8.6 to 1.3)	0.14
Higher SDM-9 score^d^	Grief	−0.1 (−0.2 to 0.0)	**0.02**
	Post-traumatic stress	−0.1 (−0.2 to 0.0)	**0.01**
	Mental health	0.0 (−0.1 to 0.1)	0.51
Higher SWD score^e^	Grief	−0.8 (−1.2 to −0.3)	**0.002**
	Post-traumatic stress	−0.4 (−0.8 to 0.0)	0.06
	Mental health	−0.1 (−0.5 to 0.2)	0.46
More days affected by pretermination HRQOL^f^	Grief	0.1 (−0.2 to 0.4)	0.47
	Post-traumatic stress	0.0 (−0.2 to 0.3)	0.87
	Mental health	0.2 (0.0 to 0.4)	**0.04**
Higher anxiety score (STAI)^g^	Grief	0.8 (0.7 to 0.9)	**<0.0001**
	Post-traumatic stress	0.5 (0.3 to 0.6)	**<0.0001**
	Mental health	0.2 (−0.1 to 0.4)	**0.01**
Higher depression score (PHQ-9)^h^	Grief	−0.1 (−0.7 to 0.5)	0.72
	Post-traumatic stress	−0.1 (−0.6 to 0.3)	0.61
	Mental health	−0.1 (−0.5 to 0.3)	0.71
Higher self-judgment score	Grief	7.4 (5.1 to 9.4)	**<0.0001**
	Post-traumatic stress	4.3 (2.1 to 6.4)	**<0.0001**
	Mental health	2.3 (0.4 to 4.1)	**0.02**
Higher community condemnation score	Grief	6.1 (2.2 to 9.8)	**0.02**
	Post-traumatic stress	5.2 (1.9 to 8.5)	**0.02**
	Mental health	0.1 (−2.7 to 3.0)	0.94

^a^ Grief measured by PGS, range = 19–95, higher scores indicate more grief.

^b^ Post-traumatic stress measured by IES, range = 0–55, higher scores indicate more post-traumatic stress or poorer coping.

^c^ Mental health (HRQOL), range = 1–30, higher scores indicate more days of activity affected by poor mental health.

^d^ SDM-9, range = 0–100, higher scores indicate higher shared decision-making.

^e^ SWD, range = 6–30, higher scores indicate higher satisfaction.

^f^ Mental health (HRQOL), range = 1–30, higher scores indicate more days of activity affected by poor mental health.

^g^ STAI, range = 20–80, higher scores indicate more anxiety.

^h^ PHQ-9, range = 0–27, higher scores indicate more depression.

*p* < 0.05.

For present-day grief, the final adjusted analysis controlled for current income, current SWD, and trait anxiety. In the adjusted model for present-day post-traumatic stress, we controlled for trait anxiety and current SWD; and in the adjusted model for self-reported mental health, we controlled for education level, trait anxiety, and preabortion mental health. In the adjusted models, self-judgment at the time of abortion was significantly associated with increased grief (*β* = 2.4, *p* = 0.02) and was not associated with differences in post-traumatic stress or postabortion mental health (Table 3).

**Table 3.** Adjusted Linear Regression of Postabortion Grief, Coping, and Mental Health-Related Quality of Life

Dependent variable	Independent variable	Regression coefficient (95% CI)	*p*
Grief (*n* = 60)	Self-judgment	2.4 (0.4 to 4.5)	**0.02**
	Income	−0.4 (−4.6 to 3.8)	0.84
	Satisfaction with decision	−0.2 (−0.5 to 0.1)	0.17
	Anxiety	0.7 (0.5 to 0.8)	**<0.001**
	Community condemnation	−0.7 (−3.3 to 2.0)	0.60
	Income	−1.2 (−5.6 to 3.2)	0.58
	Satisfaction with decision	−0.3 (−0.6 to 0.0)	0.07
	Anxiety	0.8 (0.6 to 0.9)	**<0.001**
Post-traumatic stress (*n* = 60)	Self-judgment	0.7 (−1.9 to 3.3)	0.58
	Shared decision-making	−0.1 (−0.2 to 0.0)	0.19
	Anxiety	0.4 (0.2 to 0.6)	**<0.001**
	Having a graduate degree	−1.9 (−6.8 to 2.9)	0.43
	Community condemnation	0.4 (−2.9 to 3.6)	0.83
	Shared decision-making	−0.1 (−0.2 to 0.0)	0.18
	Anxiety	0.5 (0.3 to 0.7)	**<0.001**
	Having a graduate degree	−2.3 (−7.1 to 2.5)	0.34
Mental HRQOL (*n* = 70)	Self-judgment	2.0 (0.0 to 4.1)	**0.05**
	Having a graduate degree	7.5 (3.6 to 11.4)	**<0.001**
	Preabortion mental health	0.2 (0.0 to 0.4)	**0.03**
	Anxiety	0.1 (0.0 to 0.3)	0.11
	Community condemnation	−0.7 (−3.5 to 2.1)	0.60
	Having a graduate degree	6.8 (2.7 to 10.8)	**<0.01**
	Preabortion mental health	0.2 (0.0 to 0.4)	**0.02**
	Anxiety	0.2 (0.1 to 0.4)	**0.01**

*p* < 0.05.

**FIG. 1.**
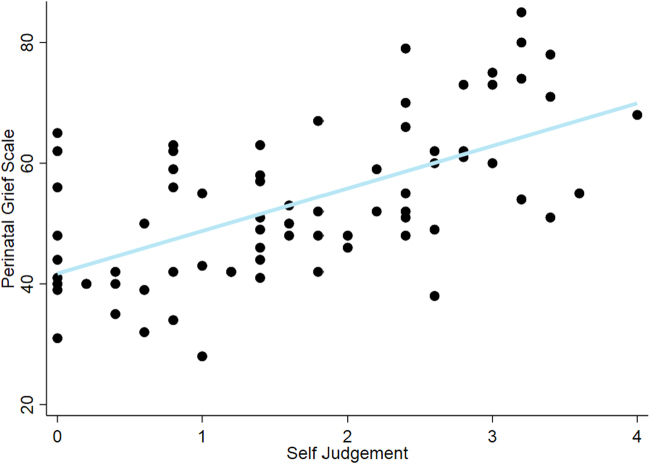
Adjusted linear regression depicting the relationship between self-judgment and grief.

Higher self-judgment at the time of abortion was associated with increased present-day grief (Fig. 1). We observed no association between perception of community condemnation at the time of abortion and these psychological outcomes in the adjusted models. Trait anxiety remained significant in all models except that modeling the relationship between self-judgment and mental HRQOL (Table 3).

When comparing adjusted models with and without anxiety as a covariate, we found that the association between self-judgment and post-traumatic stress disappeared when we included anxiety in the model (Supplementary Table S2). This sensitivity analysis with anxiety as a covariate did not affect the association between stigma and other outcomes.

## Discussion

In this cross-sectional study of women who had an abortion between 2010 and 2015, self-judgment at the time of abortion was associated with more present-day grief, but not with statistically significant differences in post-traumatic stress or mental HRQOL. We build on an emerging body of literature examining associations between abortion stigma and psychological outcomes postabortion. Our findings suggest that interventions aimed at reducing self-judgment could improve postabortion grief outcomes in this population.

Data consistently show that undergoing abortion itself is not associated with negative psychological outcomes postabortion^30,31^; however, there is mounting evidence that abortion stigma contributes to psychological distress. As mentioned in the Introduction section, Hanschmidt et al. found that in women who underwent abortion for fetal anomalies in Germany, a composite measure of abortion stigma was associated with grief, post-traumatic stress, and depression postabortion.^10^

The reported associations between stigma and grief are consistent with our findings. However, self-judgment was not associated with more post-traumatic stress in our population. Furthermore, we added postabortion mental HRQOL to our outcomes to better understand how stigma impacts a woman's general perception of her own mental health. In our study population, we did not find a statistically significant association between stigma and mental HRQOL.

Similarly, a cross-sectional study conducted in Ireland found that internalized stigma (also referred to as self-judgment) and stigma-related isolation are associated with higher levels of psychological distress, as measured by the Hospital Anxiety and Depression Scale. Two important differences between our studies were that abortion was illegal at the time of the Ireland study and they did not solely focus on abortion for maternal or fetal complications.^11^

Our finding that internalized stigma (or self-judgment) may be associated with increased postabortion grief is consistent with the findings of the Ireland study, as is our finding that perception of community condemnation was not associated with psychological outcomes. These comparisons are difficult, however, since the Ireland study did not solely include abortions for complications. We found that self-judgment as a predictor of post-traumatic stress was sensitive to trait anxiety—that is, when controlling for anxiety, there was no association between self-judgment and post-traumatic stress.

This follows logically from the fact that individuals with higher trait anxiety experience and report more negative emotions. To our knowledge, prior studies of the association between stigma and psychological outcomes among people terminating for a fetal anomaly have not explored anxiety as a confounder.

One hypothesis for not finding an association between perception of community condemnation and psychological factors is that abortion in the setting of fetal anomalies may impart less stigma than abortion for other reasons. In fact, Mosley et al. found that abortion stigma is highly dependent on intersectional factors and the local context. Results of this 2019 study suggest that in the US, abortion in the setting of fetal anomalies is more socially acceptable, which is consistent with our finding that more perceived community condemnation is not associated with grief, post-traumatic stress, or mental HRQOL.^32^

Because conceptions of stigma are often driven by local political and cultural factors,^1,31,33^ it is critical to examine this phenomenon in the population of interest. Biggs et al. found in a 928-person longitudinal study conducted in the US that most people considering abortion perceive abortion stigma and that this stigma is associated with future psychological distress.^2^ This finding further supports the association we found between abortion stigma and postabortion mental health. The trends identified in our unadjusted analyses are also consistent with previous studies.^10,20^

Geographical diversity and use of validated instruments are major strengths of this study. Specifically, our ability to control for trait anxiety and SWD is a major difference between our study and prior studies, which adds strength to our analysis of the association between abortion stigma and psychological outcomes. Many studies that have found inaccurate associations between abortion and poor mental health outcomes fail to examine whether and how present-day mental health or trait qualities may impact reporting on prior events.^34^

In our unadjusted analyses, trait anxiety was associated with all three of the psychological outcomes we examined. Thus, trait anxiety was included in our final model to control for recall bias derived from trait anxiety. As individuals with higher trait anxiety have a tendency to experience and report more negative emotions, it is possible that inclusion of trait anxiety in our model partially explains why the univariate association between self-judgment and post-traumatic stress is not statistically significant in the multivariate model.

Similarly, the fact that we controlled for present-day SWD reduces recall bias that might result from changing feelings about the abortion being reflected upon. However, we did not collect information on anxiety and depression before abortion; only information on overall perception of mental health preabortion using the HRQOL-3 was collected. While we believe overall mental health is a useful indicator, the ability to parse out effects of anxiety and depression at the time of abortion would have made for a stronger analysis.

Despite our ability to control for trait anxiety, present-day depression, and SWD, the changing political climate and varying levels of accuracy of recall could impact our results. This may be particularly true for HRQOL measures, which ask participants to recall the number of days affected by poor mental health pre- and postabortion.

Finally, because of the cross-sectional design, there is potential for reverse causality in this study—that is, present-day grief makes it more likely for subjects to report higher self-judgment at the time of abortion. In addition, while we believe it is more plausible that stigma, a deeply embedded and well-studied experience, leads to negative psychological outcomes rather than the opposite, our study design does not allow for this interpretation.

The small sample size and lack of racial, ethnic, and socioeconomic diversity limit its generalizability to the overall population of women who undergo abortion in the US.^35^ Because we used a convenience sample and data are missing for some variables, our results could be affected by selection bias. One possible impact of selection bias is that women who experienced severe postabortion psychological distress or abortion stigma may not have wanted to participate in this study, which would have led to an artificially low effect size.

Future research should include a larger and more representative sample of women seeking second-trimester abortion for maternal or fetal complications with attention to how intersectional racial and ethnic identities affect perceived stigma. Closer examination of predictors of abortion stigma is important for further understanding of how to build interventions for reducing perceived stigma.

Given the growing number of state-level gestational age restrictions on abortion in the US, particular attention to how legal restrictions on abortion impact stigma is warranted.^36^

## Conclusions

While data consistently show that abortions are not associated with poor mental health outcomes, abortion stigma, particularly self-judgment, may contribute to increased perinatal grief following abortion. Investigating how different counseling methods impact levels of self-judgment could inform the development of interventions aimed at improving grief outcomes postabortion.

Abortion providers may benefit from training to identify women who display signs of self-judgment since these women may be at higher risk for experiencing grief after abortion due to pregnancy complications. Identifying women at higher risk for poorer outcomes offers opportunities to provide additional support pre- and postabortion.

## Supplementary Material

Supplemental data

Supplemental data

## Abbreviations Used

D&E: dilation and evacuation
GED: General equivalency diploma
HRQOL: health-related quality of life
IES: Impact of Event Scale
ILAS: Individual-Level Abortion Stigma
PGS: Perinatal Grief Scale
PHQ-9: Patient Health Questionnaire 9
SD: standard deviation
SDM-9: shared decision-making
STAI: State-Trait Anxiety Inventory
SWD: Satisfaction with Decision
US: United States

## References

[1] Kumar A, Hessini L, Mitchell EMH. Conceptualising abortion stigma. Cult Health Sex 2009;11:625–639. 19437175 10.1080/13691050902842741

[2] Biggs MA, Brown K, Foster DG. Perceived abortion stigma and psychological well-being over five years after receiving or being denied an abortion. PLoS One 2020;15:e0226417. 31995559 10.1371/journal.pone.0226417 PMC6988908

[3] Shellenberg KM, Tsui AO. Correlates of perceived and internalized stigma among abortion patients in the USA: An exploration by race and Hispanic ethnicity. Int J Gynecol Obstet 118(S2):S152–S159. 10.1016/S0020-7292(12)60015-0 22920620

[4] Crane JP, LeFevre ML, Winborn RC, et al. A randomized trial of prenatal ultrasonographic screening: Impact on the detection, management, and outcome of anomalous fetuses. The RADIUS Study Group. Am J Obstet Gynecol 1994;171:392–399. 10.1016/s0002-9378(94)70040-0 8059817

[5] Nelson K, Holmes LB. Malformations due to presumed spontaneous mutations in newborn infants. N Engl J Med 1989;320:19–23. 2909875 10.1056/NEJM198901053200104

[6] Shulman LP, Grevengood C, Phillips OP, Gross SJ, Mace PC, Elias S. Family planning decisions after prenatal detection of fetal abnormalities. Am J Obstet Gynecol 1994;171:1373–1376. 7977549 10.1016/0002-9378(94)90163-5

[7] Reasons U.S. Women Have Abortions: Quantitative and Qualitative Perspectives [Internet]. Guttmacher Institute, 2005. Available at: https://www.guttmacher.org/journals/psrh/2005/reasons-us-women-have-abortions-quantitative-and-qualitative-perspectives Accessed March 9, 2022.

[8] Kerns J, Vanjani R, Freedman L, Meckstroth K, Drey EA, Steinauer J. Women's decision making regarding choice of second trimester termination method for pregnancy complications. Int J Gynaecol Obstet Off Organ Int Fed Gynaecol Obstet 2012;116:244–248. 10.1016/j.ijgo.2011.10.016 22169044

[9] Maguire M, Light A, Kuppermann M, Dalton VK, Steinauer JE, Kerns JL. Grief after second-trimester termination for fetal anomaly: A qualitative study. Contraception 2015;91:234–239. 25499590 10.1016/j.contraception.2014.11.015 PMC4406975

[10] Hanschmidt F, Treml J, Klingner J, Stepan H, Kersting A. Stigma in the context of pregnancy termination after diagnosis of fetal anomaly: Associations with grief, trauma, and depression. Arch Womens Ment Health 2018;21:391–399. 29288285 10.1007/s00737-017-0807-9

[11] O'Donnell AT, O'Carroll T, Toole N. Internalized stigma and stigma-related isolation predict women's psychological distress and physical health symptoms post-abortion. Psychol Women Q 2018;42:220–234.

[12] Korenromp MJ, Page-Christiaens GCML, van den Bout J, Mulder EJH, Visser GHA. Adjustment to termination of pregnancy for fetal anomaly: A longitudinal study in women at 4, 8, and 16 months. Am J Obstet Gynecol 2009;201:160.e1–e7. 19560116 10.1016/j.ajog.2009.04.007

[13] Korenromp MJ, Christiaens GCML, Bout J van den, et al. Long-term psychological consequences of pregnancy termination for fetal abnormality: A cross-sectional study. Prenat Diagn 2005;25:253–260. 15791682 10.1002/pd.1127

[14] Lafarge C, Mitchell K, Fox P. Termination of pregnancy for fetal abnormality: A meta-ethnography of women's experiences. Reprod Health Matters 2014;22:191–201. 10.1016/S0968-8080(14)44799-2 25555776

[15] Lafarge C, Mitchell K, Fox P. Perinatal grief following a termination of pregnancy for foetal abnormality: The impact of coping strategies. Prenat Diagn 2013;33:1173–1182. 23943597 10.1002/pd.4218

[16] Cockrill K, Upadhyay UD, Turan J, Greene Foster D. The Stigma of Having an Abortion: Development of a Scale and Characteristics of Women Experiencing Abortion Stigma [Internet]. Guttmacher Institute, 2013. Available at: https://www.guttmacher.org/journals/psrh/2013/05/stigma-having-abortion-development-scale-and-characteristics-women 10.1363/4507913 23750622

[17] Kersting A, Kroker K, Steinhard J, et al. Psychological impact on women after second and third trimester termination of pregnancy due to fetal anomalies versus women after preterm birth—A 14-month follow up study. Arch Womens Ment Health 2009;12:193–201. 19266250 10.1007/s00737-009-0063-8

[18] Salvesen KA, Oyen L, Schmidt N, Malt UF, Eik-Nes SH. Comparison of long-term psychological responses of women after pregnancy termination due to fetal anomalies and after perinatal loss. Ultrasound Obstet Gynecol Off J Int Soc Ultrasound Obstet Gynecol 1997;9:80–85. 10.1046/j.1469-0705.1997.09020080.x 9132260

[19] Gelman A, Rosenfeld EA, Nikolajski C, Freedman LR, Steinberg JR, Borrero S. Abortion stigma among low-income women obtaining abortions in Western Pennsylvania: A qualitative assessment. Perspect Sex Reprod Health 2017;49:29–36. 27984674 10.1363/psrh.12014 PMC5572656

[20] Kerns JL, Mengesha B, McNamara BC, Cassidy A, Pearlson G, Kuppermann M. Effect of counseling quality on anxiety, grief, and coping after second-trimester abortion for pregnancy complications. Contraception 2018;97:520–523. 29477632 10.1016/j.contraception.2018.02.007

[21] Potvin L, Lasker J, Toedter L. Measuring grief: A short version of the perinatal grief scale. J Psychopathol Behav Assess 1989;11:29–45.

[22] Sundin EC, Horowitz MJ. Horowitz's impact of event scale evaluation of 20years of use. Psychosom Med 2003;65:870–876. 14508034 10.1097/01.psy.0000084835.46074.f0

[23] HRQOL-14—Healthy Days Measure | HRQOL | CDC [Internet], 2018. Available at: https://www.cdc.gov/hrqol/hrqol14_measure.htm

[24] Steinberg JR, Tschann JM, Furgerson D, Harper CC. Psychosocial factors and pre-abortion psychological health: The significance of stigma. Soc Sci Med 1982 2016;150:67–75. 10.1016/j.socscimed.2015.12.007 PMC4737478 26735332

[25] Scholl I, Kriston L, Dirmaier J, Buchholz A, Härter M. Development and psychometric properties of the Shared Decision Making Questionnaire—Physician version (SDM-Q-Doc). Patient Educ Couns 2012;88:284–290. 22480628 10.1016/j.pec.2012.03.005

[26] Holmes-Rovner M, Kroll J, Schmitt N, et al. Patient satisfaction with health care decisions: The satisfaction with decision scale. Med Decis Mak Int J Soc Med Decis Mak 1996;16:58–64. 10.1177/0272989X9601600114 8717600

[27] Kroenke K, Spitzer RL, Williams JB. The PHQ-9: Validity of a brief depression severity measure. J Gen Intern Med 2001;16:606–613. 11556941 10.1046/j.1525-1497.2001.016009606.x PMC1495268

[28] Spielberger CD, Gorsuch RL, Lushene R, Vagg PR, Jacobs GA. Manual for the state-trait anxiety inventory. Consulting Psychologists Press, Palo Alto, CA: 1983.

[29] Gidron Y. Trait Anxiety. In: Gellman MD, Turner JR, eds. Encyclopedia of behavioral medicine [Internet]. New York, NY: Springer, 2013:1989–1989. Available at: 10.1007/978-1-4419-1005-9_1539

[30] Biggs MA, Upadhyay UD, McCulloch CE, Foster DG. Women's mental health and well-being 5 years after receiving or being denied an abortion: A prospective, longitudinal cohort study. JAMA Psychiatry 2017;74:169–178. 27973641 10.1001/jamapsychiatry.2016.3478

[31] Major B, Appelbaum M, Beckman L, Dutton MA, Russo NF, West C. Abortion and mental health: Evaluating the evidence. Am Psychol 2009;64:863–890. 19968372 10.1037/a0017497

[32] Mosley EA, Anderson BA, Harris LH, Fleming PJ, Schulz AJ. Attitudes toward abortion, social welfare programs, and gender roles in the U.S. and South Africa. Crit Public Health 2020;30:441–456. 35368244 10.1080/09581596.2019.1601683 PMC8975127

[33] Norris A, Bessett D, Steinberg JR, Kavanaugh ML, De Zordo S, Becker D. Abortion stigma: A reconceptualization of constituents, causes, and consequences. Womens Health Issues Off Publ Jacobs Inst Womens Health 2011;21(3 Suppl.):S49–S54. 10.1016/j.whi.2011.02.010 21530840

[34] Charles VE, Polis CB, Sridhara SK, Blum RW. Abortion and long-term mental health outcomes: A systematic review of the evidence. Contraception 2008;78:436–450. 19014789 10.1016/j.contraception.2008.07.005

[35] Characteristics of U.S. Abortion Patients in 2014 and Changes Since 2008 [Internet]. Guttmacher Institute, 2016. Available at: https://www.guttmacher.org/report/characteristics-us-abortion-patients-2014

[36] Hall KS, Redd S, Narasimhan S, et al. Abortion trends in georgia following enactment of the 22-week gestational age limit, 2007–2017. Am J Public Health 2020;110:1034–1038. 10.2105/AJPH.2020.305653 PMC7287556 32437279

