# Supplementary material for: Abortion Stigma and Its Relationship with Grief, Post-traumatic Stress, and Mental Health-Related Quality of Life After Abortion for Fetal Anomalies
Source: Womens Health Rep (New Rochelle). 2022 Mar 28;3(1):385–94. doi: 10.1089/whr.2021.0027 (PMC8994429; doi:10.1089/whr.2021.0027)
Supplement: Supplemental data [file Suppl_TableS1.docx]

Supplemental Table 1. Demographic comparison between people who completed predictors and outcomes of interest and those who did not.

| **Characteristic** | **Complete Data (N=80)** | **Incomplete Data (N=33)** |
| --- | --- | --- |
| Age at termination, years, mean (SD)  Missing, N (%) | 35.5 (5.2)  0 (0.0) | 36.1 (9.5)  0 (0.0) |
| Race/ethnicity, N (%)  White  Non-white  Missing | 70 (87.5)  10 (12.5)  0 (0.0) | 10 (30.3)  23 (69.7)  0 (0.0) |
| Annual income, N (%)  Greater than $90,000  Less than $90,000  Missing | 54 (67.5)  26 (32.5)  0 (0.0) | 8 (24.2)  3 (9.1)  22 (66.7) |
| Highest level of education, N (%)  Less than high school  High School or GED  Bachelor’s degree  Graduate degree  Missing | 1 (1.3)  1 (1.3)  39 (48.8)  39 (38.8)  0 (0.0) | 0 (0.0)  1 (3.0)  6 (18.2)  4 (12.1)  22 (66.7) |
| Urban-rural classification, N (%)  Urban  Suburban  Rural  Missing | 27 (33.8)  46 (57.5)  5 (6.3)  2 (2.5) | 7 (21.1)  3 (9.1)  1 (3.0)  22 (66.7) |
| Insurance type at time of study, N (%)  Private  Public  None  Missing | 79 (98.8)  0 (0.0)  1 (1.2)  0 (0.0) | 9 (27.3)  1 (3.0))  0 (0.0)  23 (69.7) |
| Gestational duration (in wks) at abortion, mean (SD)  Missing, N (%) | 20.5 (3.8)  0 (0.0) | 19.1 (3.4)  3 (0.1) |
| Abortion procedure type, N (%)  Dilation and evacuation  Induction of labor  Not specified | 52 (65.0)  20 (24.4)  8 (10.0) | 9 (27.3)  7 (21.2)  17 (51.5) |
| Region of United States where abortion occurred, N (%)  West  Northeast  Midwest  South  Missing | 43 (53.8)  13 (16.3)  7 (8.8)  17 (21.3)  0 (0.0) | 22 (66.7)  1 (3.0)  5 (15.2)  5 (15.2)  0 (0.0) |
| Nulliparous, N (%)  Yes  No  Missing | 31 (38.8)  48 (60.0)  1 (1.2) | 9 (27.3)  20 (60.6)  4 (12.1) |
| Reason for abortion, N (%)  Fetal complications  Maternal complications  Missing | 4.9 (3.7)  8 (10.0) | 16 (48.5)  1 (3.0)  16 (48.5) |
| Wait time from decision about procedure type to abortion, days, mean (SD)  Missing, N (%) | 62.5 (27.1)  8 (10.0) | 4.6 (2.4)  24 (72.7) |
| Shared Decision Making (SDM-9)^a^, score, mean (SD)  Missing, N (%) | 22.6 (6.0)  8 (10.0) | 58.2 (30.9)  28 (84.9) |
| Satisfaction with Decision (SWD)^b^, score, mean (SD)  Missing, N (%) | 1.7 (1.1)  8 (10.0) | 21 (2.6)  30 (90.9) |
| Self-judgement^c^, score, mean (SD)  Missing, N (%) | 1.4 (0.8)  10 (12.5) | --  -- |
| Community condemnation^d^, score, mean (SD)  Missing, N (%) | 43.9 (13.6)  2 (2.5) | --  -- |
| Anxiety (STAI)^e^, score, mean (SD)  Missing, N (%) | 8.0 (5.6)  6 (7.5) | --  -- |
| Depression (PHQ9)^f^, score, mean (SD)  Missing, N (%) | 57.5 (13.2)  1 (1.3) | --  -- |
| Grief (PGS)^g^, score, mean (SD)  Missing, N (%) | 24.3 (10.9)  2 (2.5) | --  -- |
| Coping (IES)^h^, score, mean (SD)  Missing, N (%) | 10.7 (10.3)  6 (7.5) | --  -- |
| Mental health^i^ pre-abortion, days, mean (SD)  Missing, N (%) | 24.6 (9.1)  6 (7.5) | --  -- |
| Mental health^i^ post-abortion, days, mean (SD)  Missing, N (%) | 35.5 (5.2)  0 (0.0) | --  -- |

^a^SDM-9 range = 0-100, higher scores indicate higher shared decision making

^b^SWD range = 6-30, higher scores indicate higher satisfaction

^c^Self-judgement range = 0-4, higher scores indicate more self-judgement

^d^Community condemnation range = 0-4, higher scores indicate more feelings of community condemnation

^e^STAI range = 20-80, higher scores indicate more anxiety

^f^PHQ9 range = 0-27, higher scores indicate more depression

^g^PGS range = 19-95, higher scores indicate more grief

^h^IES range = 0-55, higher scores indicate more posttraumatic stress or poorer coping

^i^Mental health (HRQOL) range = 1-30, higher scores indicate more days of activity affected by poor mental health
