# Supplementary material for: Abortion Stigma and Its Relationship with Grief, Post-traumatic Stress, and Mental Health-Related Quality of Life After Abortion for Fetal Anomalies
Source: Womens Health Rep (New Rochelle). 2022 Mar 28;3(1):385–94. doi: 10.1089/whr.2021.0027 (PMC8994429; doi:10.1089/whr.2021.0027)
Supplement: Supplemental data [file Suppl_TableS2.docx]

Supplemental Table 2. Sensitivity Analysis for Trait Anxiety

| **Dependent Variable** | **Independent Variable** | **Original Analysis** | | **Sensitivity Analysis** | |
| --- | --- | --- | --- | --- | --- |
|  |  | **Regression Coefficient (95% CI)** | **P value** | **Regression Coefficient (95% CI)** | **P value** |
| Grief (n=60) | **Self-judgement**  Income  Satisfaction with decision  Anxiety | 2.4 (0.4, 4.5)  -0.4 (-4.6, 3.8)  -0.2 (-0.5, 0.1)  0.7 (0.5, 0.8) | **0.02**  0.84  0.17  **<0.001** | 6.7 (4.1, 9.3)  -1.8 (-8.0, 4.4)  -0.5 (-0.9, 0.0)  -- | **<0.001**  0.56  0.44  -- |
|  | **Community condemnation**  Income  Satisfaction with decision  Anxiety | -0.7 (-3.3, 2.0)  -1.2 (-5.6, 3.2)  -0.3 (-0.6, 0.0)  0.8 (0.6, 0.9) | 0.60  0.58  0.07  **<0.001** | 4.0 (-0.3, 8.0)  -3.4 (-10.5, 3.7)  -0.7 (-1.2, -0.1)  -- | 0.52  0.35  **0.01**  -- |
| Posttraumatic stress (n=60) | **Self-judgement**  Shared decision making  Anxiety  Having a graduate degree | 0.7 (-1.9, 3.3)  -0.1 (-0.2, 0.0)  0.4 (0.2, 0.6)  -1.9 (-6.8, 2.9) | 0.58  0.19  **<0.001**  0.43 | 3.6 (1.1, 6.1)  -0.1 (-0.2, 0.0  --  -1.0 (-6.3, 4.3) | **0.005**  0.12  --  0.71 |
|  | **Community condemnation**  Shared decision making  Anxiety  Having a graduate degree | 0.4 (-2.9, 3.6)  -0.1 (-0.2, 0.0)  0.5 (0.3, 0.7)  -2.3 (-7.1, 2.5) | 0.83  0.18  **<0.001**  0.34 | 3.6 (-0.0, 7.2)  -0.1 (-0.2, 0.0)  --  -2.5 (08.1, 3.2) | 0.053  0.07  --  0.39 |
| Mental health-related quality of life (n=70) | **Self-judgement**  Having a graduate degree  Pre-abortion mental health  Anxiety | 2.0 (0.0, 4.1)  7.5 (3.6, 11.4)  0.2 (0.0, 0.4)  0.1 (0.0, 0.3) | **0.05**  **<0.001**  **0.03**  0.11 | 2.9 (1.2, 4.7)  7.4 (3.6, 11.3)  0.2 (0.0, 0.4)  -- | **0.001**  **<0.001**  **0.02**  -- |
|  | **Community condemnation**  Having a graduate degree  Pre-abortion mental health  Anxiety | -0.7 (-3.5, 2.1)  6.8 (2.7, 10.8)  0.2 (0.0, 0.4)  0.2 (0.1, 0.4) | 0.60  **<0.01**  **0.02**  **0.01** | 0.6 (-2.1, 3.3)  6.2 (2.0, 10.3)  0.25 (0.0, 0.5)  -- | 0.66  **<0.01**  **0.01**  -- |
